# Design and preliminary evaluation of a track-based robotic colonoscope with a shape-adaptable tip for propulsion

**DOI:** 10.3389/frobt.2025.1580692

**Published:** 2025-04-14

**Authors:** Jiayang Du, Lin Cao, Sanja Dogramadzi

**Affiliations:** School of Electrical and Electronic Engineering, The University of Sheffield, Sheffield, United Kingdom

**Keywords:** robotic endoscope, expansion mechanism, track-based, tracked, selfpropelling

## Abstract

This paper introduces a shape-adaptable robotic endoscope design, which combines an expansion mechanism and external drive system that provide tip insertion force and adjust the tip shape and size to different colon diameters. Expansion rate of 53% has been achieved in the expandable tip size, which corresponds to changes in the colon diameter. We tested the prototype locomotion in a pipe with different friction surface layers, including artificial bowel tissues, to assess propulsion force and normal force on the colon that can be achieved with the current design. The prototype can generate a propulsion force of 2.83 N, and the maximum linear speed of 29.29 mm/s on the artificial tissue surface. It can produce effective propulsion when it passes through pipes of different diameters. The results demonstrate the prototype’s ability for shape adaptation that maintains the required traction force on the bowel wall.

## 1 Introduction

The last few decades have witnessed significant transformation in human behavioural patterns and nutritional profiles, characterized by increased occupational sedentarism, insufficient dietary fibre consumption, and chronic psychosocial stressors (Nagase et al., 2018). These have led to a sharp increase in the incidence of colon-related diseases, with colorectal carcinoma demonstrating the most pronounced correlation (Carrozza et al., 1996).

While colonoscopy remains the gold-standard modality for both diagnostic visualization and therapeutic intervention within the colonic lumen, population-scale implementation faces three primary challenges: (1) procedural discomfort affecting patient compliance, (2) operator-dependent detection accuracy, and (3) limited healthcare resource availability. Although clinical evidence confirms that routine endoscopic screening reduces colorectal cancer incidence through adenoma detection and removal (Valdastri et al., 2012a; Valdastri et al., 2012b), current methodology demonstrates suboptimal practicality and acceptability as a mass screening tool.

During the procedure, clinicians maintain bimanual dexterity; one hand regulates the insertion parameters, such as depth, velocity, and propulsion force, through manual torque transmission, while the other hand manipulates two dials to dominate the semi-flexible distal tip. Patient acceptance rates for colonoscopy remain a persistent clinical challenge (Chen et al., 2019), as the procedure entails inherent risks including looping of the colonoscope, intestinal distension, procedural discomfort, and in rare instances, bowel perforation (Appleyard et al., 2000), all of which may result in physical trauma and psychological distress. Moreover, colonoscopy clinical training is rigorous and often exhausting, which can result in repeated strain injuries to the hand and wrist muscles and ligaments (Valdastri et al., 2012). All of these have served as compelling incentives for robotics researchers to find a more efficient and less painful way to perform colon inspection and treatment.

Given the drawbacks of traditional colonoscopies, there is a strong shift towards developing autonomous robotic systems. These advanced technologies are designed not only to enhance the precision of colonoscopies but also to minimize discomfort and potential complications, thereby improving the overall safety and efficacy of the procedure.

Many robotic platforms for colonoscopy have been proposed in the last couple of decades. Since the introduction of the miniature ingestible capsules with a camera for recording the colon internal wall in 2000, the focus has been on their controlled propulsion [rather than peristalsis (Wang et al., 2013; Ciuti et al., 2020)] for detailed screening of colon (Kim et al., 2014; Lee et al., 2016). Gregory and others have designed endoscope equipment that drives the crawler to move through the meshing of worms gears, but improving movement efficiency remains an urgent issue (Formosa et al., 2019). Some studies have shown that for robots that relying on crawling or wheel movement, the contact area on their abdomen (underside) is not consistently maintained. This inconsistency results in insufficient traction force, impeding the robot’s effective propulsion (Hosoe et al., 2019; Nakamura et al., 2012). Therefore, a robotic colonoscope based on wall-pressing has begun to attract attention (Norton et al., 2016) (Mills et al., 2017). Several attempts have been made to propel a capsule equipped with an internal magnetic coil using a set of external magnets (Valdastri et al., 2012; Verra et al., 2020). However, the complexity of the human tortuous colon and the friction forces between the capsule and the colon often prevent this interesting approach from achieving complete success (Chen et al., 2022). Robotic colonoscopes have also been inspired by the locomotion of inchworms, snakes, and centipedes design to propagate through the colon. Those robots use effectors that require multiple continuous contacts with the colon wall for anchoring, traction, or footholds. Reliable anchoring of robots in the colonic lumen continues to be a critical challenge (Ciuti et al., 2020). Moreover, while gaining sufficient traction and foothold to propel the robot forward is necessary (Nagase et al., 2018; Kim et al., 2014;Lee et al., 2016), it can also result in tissue damage. A recent work on a traction-based robot mechanism that consists of two independent balloons and a transmission system based on tracked motion (Consumi et al., 2023) has been reported to successfully navigate the colon lumen. The wall-pressing centre the endoscope head within the lumen and provide traction via several tracks that contact the colon wall. Even though the force propelling this tethered head generates tip force, its ability to steer around sharp colon angles in the colon has not been realized. Furthermore, maintaining consistent traction force throughout the colon, which varies in diameter along its 1 m length, remains a challenge.

In this paper, we present a new robotic endoscope prototype that builds on insights from previous research (Valdastri et al., 2012) and the authors’ earlier work (Dogramadzi, 2001). This prototype features an advanced expansion mechanism designed to address the challenges of radial adaptation along the entire length of the colon. The incorporation of an expansion mechanism ensures that the robot’s head treads are always under tension, facilitating continuous contact with the colon’s inner wall to obtain sufficient traction. Additionally, combining wall-pressing tread drive with the expansion mechanism allows the robot to effectively address the loss of traction that occurs due to changes in the size of the intestinal tract during navigation. The adjustable size of the expansion mechanism enables the robot to adapt to various colon sizes. Furthermore, the independently controlled expansion mechanism allows for precise control of each tread, laying the foundation for the robot’s flexible manoeuvring within the intestinal tract. This capability significantly enhances the potential for autonomous colonoscopy examinations. The contribution of this study include: 1. A kinematic analysis of the shape-morphing endoscopic robotic prototype; 2. The design and fabrication of the prototype; 3. The design and implementation of an experimental rig for testing robot locomotion and the adaptation of its tip’s diameter.

## 2 Prototype design

### 2.1 Colon diameters

The human colon is an elastic, muscular tube that enables it to effectively transport and process food residues during digestion. It consists of multiple tissue layers, such as the inner mucosa, muscular layer, and outer serosa, which provide the colon with the strength and flexibility needed to expand and contract under pressure. The diameter of the colon varies significantly along its length, reflecting its different functions. For instance, the larger diameters of the ascending colon and cecum accommodate water and electrolyte absorption, requiring more space for storing and processing contents. In contrast, the narrower sigmoid colon and rectum are primarily responsible for storing and expelling faeces, thus requiring less capacity than the ascending sections.

Discovery of human colon anatomy research reveals significant details about its structure. The human colon is a continuous hollow tube approximately 1.5 m long and 75 mm in diameter when inflated (Kim et al., 2014). It comprises six segments: the rectum, sigmoid colon, descending colon, transverse colon, ascending colon, and cecum. The average diameters of these segments range from 26 to 45 mm, as detailed in Table 1 (Quirini et al., 2008).

**TABLE 1:** Average diameter of colon segments.

Colon sections	Average diameter (mm)
Rectum	36
Sigmoid colon	26
Descending colon	33
Transverse colon	37
Ascending colon	45
Cecum	44

### 2.2 Working principles

The cylindrical shape of the robot is formed by an assembly comprising four flexible tracks and four expandable bellows. The structure of robot is segmented into four main components: 1. The power transmission system, which consists of a worm gear connected by a flexible shaft that engages with the tracks; 2. The expansion tip, featuring an expansion mechanism equipped with the four bellows and four struts; 3. The four flexible tracks that make direct contact with the colon wall; and 4. The external motor and its control system.

**FIGURE 1:**
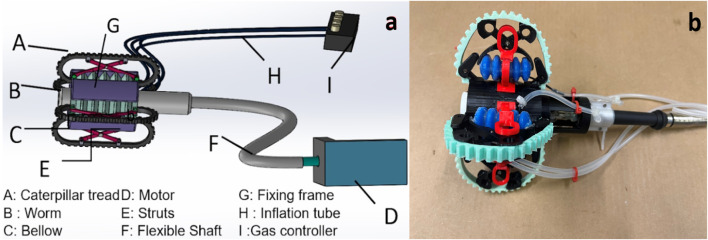
**(a)** A conceptual diagram of the robotic endoscope. The endoscope tip is driven using a worm couple **(B)** actuated by an external motor **(D)** through a flexible shaft. The tip also features four independently controlled expansion mechanisms enabled by a pneumatic bellow **(C)** and strut mechanisms **(E)**, **(b)** The expandable tip. The external motor is connected to a Arduino UNO to realize locomotion of the device (not equipped with camera lens).

The external motor transmits power to the worm gear via a flexible shaft, which in turn drives the flexible tracks to produce linear motion throughout the entire structure, as shown in Figure 1. The flexible torque shaft is composed of a metal shaft and an external rubber sleeve. The flexible shaft is widely used to transmit rotary motion and torque along curved paths, despite some power dissipation due to friction between the rotating metal shaft and the rubber sleeve. The flexible bellows, which are controlled independently, adjust the displacement of scissor-shaped rigid struts to ensure continuous contact between the tracks and the inner wall of the colon. The inflation of bellows and consequent extension of the struts dictate the normal force that the tracks exert on the inner wall of the colon. The smooth back surface of each track slides over a reserved groove in the strut. On the opposite side, the external toothed surface of the track contacts the colon wall and generates friction, propelling the entire structure through the colon. The four tracks and four flexible bellows are symmetrically positioned around a cylindrical frame, with each track wrapped around the frame and connected at the ends to form a continuous loop. The expansion mechanism adjusts the robot’s external radius to maintain traction against the colon wall by extending the bellows. This extension alters the angle of the struts, pushing the tracks outward. This symmetrical structure enables a single motor and worm gear to drive all four tracks, making it highly effective for navigating narrow spaces with variable radial dimensions. The integration of external motor control with adaptive sizing facilitates improved locomotion by allowing the robot to adapt to varying colon sizes of the colon, thus ensuring stable contact and enhanced traction. Moreover, while this design holds the potential to steer the entire mechanism through sharp bends of the colon, this paper primarily reports our preliminary work concerning the straight sections of the colon.

### 2.3 Prototype modelling

**FIGURE 2:**
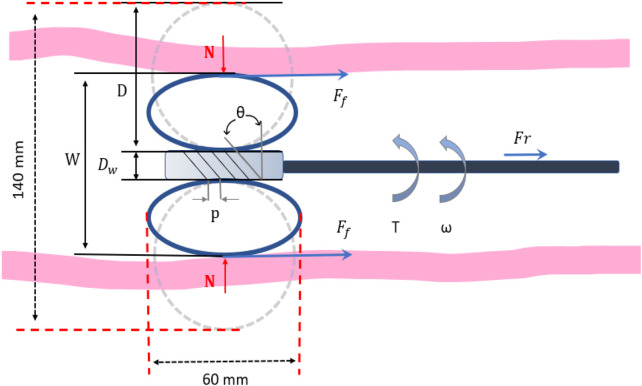
Diagram of the kinematic mechanism of the expansion tip.

We describe the motion of the robot within the colon, considering the actuation forces and friction between the tracks and colon wall, as illustrated in Figure 2. Within the colon lumen, the tracks conform to the colon diameter and exert pressure on the colon wall. For analytical purposes, our simplified model treats the colon as a rigid tube to focus on fundamental aspects of the robot’s operation, which allowing us to analyse the force exerted at the tip of the robotic colonoscope and to understand the working principle of the device’s locomotion and expansion mechanisms. This approach allows us to isolate and understand these basic dynamics before introducing more complex variables. While this model does not capture all the nuances of an actual flexible colon, it provides a necessary foundation for initial experiments. As our research advances, we plan to incorporate more detailed and realistic models that reflect the true variability and elasticity of the colon.

In this case (the normal force as the product of the track’s elastic modulus and compression), the maximum static friction between the colon wall and the track is:

$$F_{f}=n\mu_{c}N=n\mu_{c}k\left(D-\frac{W-D_{w}}{2}\right)$$ (1)

Where n denotes the number of tracks, $\mu_{c}$ is the frictional coefficient between the track and lumen wall, N is the normal force of track, k is the radial equivalent elastic modulus of track, D is the diameter, W is the diameter of colon lumen, $D_{w}$ is the diameter of the worm gear.

In the initial stages of this study, we only used the static friction model (see Equation 1) to evaluate the device’s ability to overcome starting resistance, mainly because the difference between static and dynamic friction is relatively limited during low-speed, intermittent operation; after overcoming initial inertia, the adhesion or blocking effect is not significant. However, we are fully aware that in practical applications, dynamic friction and adhesion factors cannot be ignored, and we will incorporate these effects in more complex experimental conditions to improve the evaluation of propulsion performance and safety.

The motor drives the sole worm gear, and the angular velocity of the motor can be expressed as follows (see Equation 2):

$$v=\frac{D_{w}\tan\theta \omega }{2}=\frac{\omega p}{2\pi p}$$ (2)

Where ω represents the angular velocity of the motor, p denotes the pitch of the worm, and v is the velocity of the robot.

The propelling force and torque generated by the worm gear are as follows:

$$F_{r}=Ncos\theta -µNsin\theta$$ (3)

$$T=\frac{D_{w}}{2}\left(Nsin\theta -µNcos\theta \right)+T_{f}$$ (4)

Where N is the normal force, µ is the frictional coefficient between the worm and the track, T represents the output torque of motor, and $T_{f}$ is the reaction torque (resulting from the friction between worm and tracks).

According to the equation pitch angle of worm,

$$\tan\theta =p/D_{w}\pi$$ (5)

Therefore, based on the previous Equations 3–5, the relationship between motor torque and propelling force can be derived as follows:

$$F_{r}=\frac{2\left(\pi D_{w}-\mu p\right)\left(T-T_{f}\right)}{D_{w}\left(\mu \pi D_{w}+p\right)}$$ (6)

If we assume the relationship between output torque and rotation speed is linear, it can be determined as follows:

ω=ωn1−TTM (7)

Where $\omega_{n}$ is angular velocity under no load, $T_{M}$ is maximum torque when angular velocity is zero.

Therefore, according to Equations 6 and 7, the maximum propelling force of the robot can be determined (see Equation 8):

$$F_{\text{rmax}}=\frac{2\left(\pi D_{w}-\mu p\right)\left(T_{M}-T_{f}\right)}{D_{w}\left(\mu \pi D_{w}+p\right)}$$ (8)

The worm gear transmission ratio is:

$$i=\frac{Z_{w}}{Z}$$ (9)

Where $Z_{w}$ and Z represent the number of teeth and track respectively, which are shown in Table 2.

**TABLE 2:** Specifications of the fabricated robot.

Parameter	Dimension
Worm teeth number, Z	5
Track Teeth number, $Z_{w}$	34
The prototype frame width (mm)	140
Track width (mm)	7.5
Belt height (mm)	4.5
Tooth length (mm)	3
Worm gear pitch (mm), p	3
Worm gear diameter (mm), $D_{w}$	28

The linear velocity of the worm can be calculated using:

$$v_{w}=\frac{\pi D_{w}n_{w}}{60}$$ (10)

Where $n_{w}$ represents the rotation speed of the worm gear, which corresponds to the angular speed of the motor. We neglect the sliding of the flexible shaft and rotational friction resistance in the flexible shaft. According to Equations 9, 10, the linear speed of the prototype can be expressed as follows (see Equation 11):

$$v=i\cdot v_{w}$$ (11)

### 2.4 Prototype fabrication

Considering the average dimensions of the human colon, the width of the robot’s, which measures 140 mm, is considerably larger—four times the size of the intended final model. This is because we are in the theoretical testing phase, where the aim is to validate the feasibility of design concepts and technical assumptions. At this stage, using a larger model facilitates the adjustment and testing of various mechanical components, thereby ensuring the accuracy of the concept. Although this size is not suitable for human trials at present, it provides us with essential data and feedback that help us understand the challenges that might be encountered in practical applications.

The dimensional characteristics of the current prototype are shown in Table 2.

**FIGURE 3:**
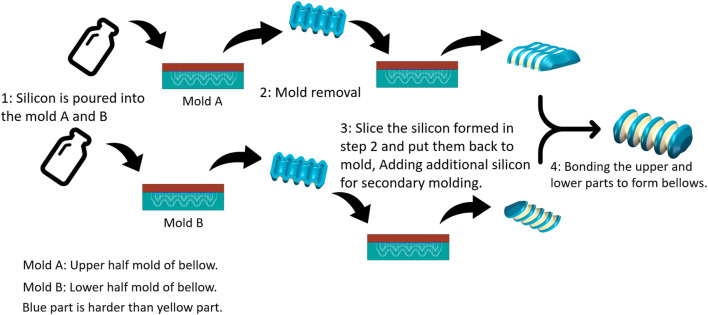
Fabrication of the soft-bodied bellows.

The actuation of the prototype is facilitated by an external stepping motor (NEMA 17, Rtelligent), which is controlled by a microcontroller (Arduino Uno). The rigid worm gear and the outer sleeve are made from PLA obtained through 3D printing. The innermost sleeve features four grooves that limit the relative sliding and lateral movements of the track. The outermost frame encases the worm and tracks. To reduce excessive friction between the tracks and the sleeve, the track groove is arc-shaped and filled with lubricant. The bellows are fabricated in two parts and then bonded together (see Figure 3).

To limit the radial deformation of the bellows and maximize axial deformation, two different silicon materials with varying hardness levels were used in their manufacturing: the blue part has a shore hardness of 30A (Mould-star, Smooth-On, United States), and the yellow part has a shore hardness of 00-30 (Eco-flex 00-30, Smooth-On, United States), as shown in Figure 3. An air pump was utilized to evacuate air from the silicon mixture, preventing bubble formation during curing. This process enhance the strength of the silicon and reduce the risk of fractures in the cavity.

**FIGURE 4:**
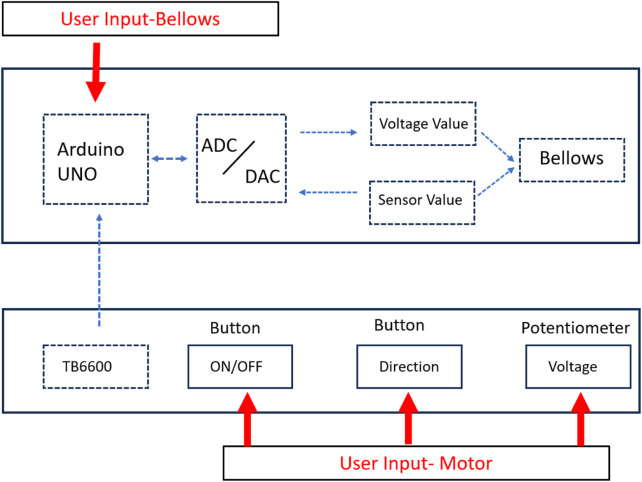
Open loop control of four independent bellows using Arduino microcontroller.

The pressure of the four independent bellows and the motor’s angular speed are adjustable by the user. In Figure 4, red characters indicate the user inputs, while blue dotted arrows indicate data communication between components in the system.

## 3 Experiment setup

### 3.1 Force and displacement measurements of bellows

**FIGURE 5:**
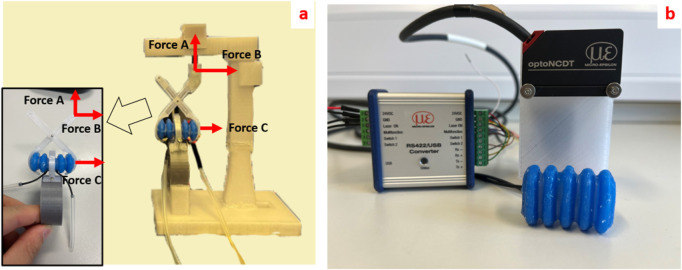
**(a)** Force sensor experimental platform of the bellow with strut (insert shows the direction and position of the force measured in the experiment); **(b)** Laser sensor platform for detecting the deformation of bellows.

**FIGURE 6:**
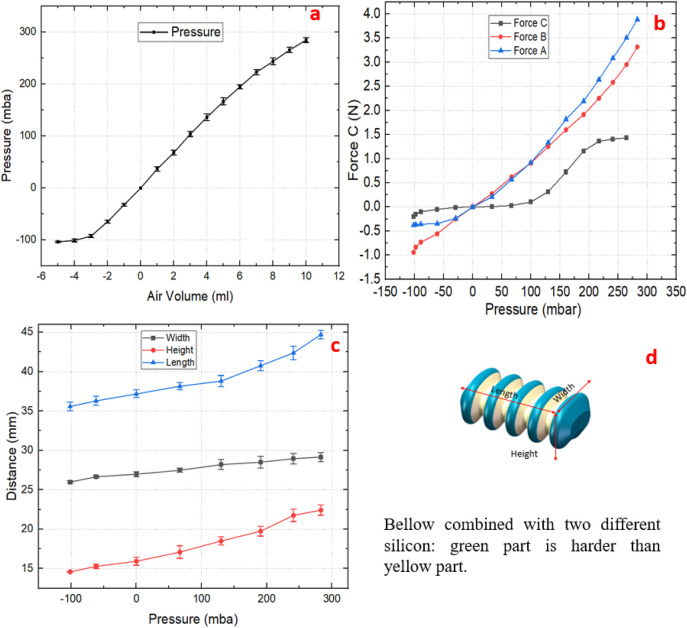
**(a)** The relationship between the air volume inside the bellows and the internal pressure; **(b)** $F_{A}$ , $F_{B}$ and $F_{C}$ as a function of pressure change in one bellow; **(c)** Displacements of the bellow along width, height and length pressure in the bellow; **(d)** Bellow rendering.

Figure 5a illustrates three forces: Force A, Force B, Force C (abbreviated as $F_{A}$ , $F_{B}$ , $F_{C}$ ) at different points of the expansion mechanism when the bellows air pressure changes. $F_{A}$ represents the normal force at the top of the strut. $F_{B}$ denotes the radial force at the same location, and $F_{C}$ is the axial force exerted by the bellow. The maximum normal bellow exerted by the bellow, $F_{A}$ is 3.89N. With the bellows cavity air volume changing from −5–10 mL, the bellows working pressure range is set to −101–283 mbar, as shown in Figure 6a. In order to evaluate the accuracy and consistency of the experimental results, 10 repeated measurements were made for each experiment without changing any experimental conditions. Each measurement is carried out independently, so as to evaluate the random error of the experiment and calculate the average and standard deviation of the data. This method allows us to obtain more reliable measurement results and better understand the random fluctuation of the experimental system.

The experimental results for the bellows displacement measurements were obtained using a laser sensor (optoNCDT 1420, MICRO EPSILON, Germany), as illustrated in Figure 5b. The maximum lengthwise displacement of the bellows is 8.98 mm. Significant changes were also observed in other dimensions, specifically 6.3 mm in the height and 2.12 mm in the width, as shown in Figure 6d. These findings demonstrate that two silicon materials of different hardness can create a flexible linear actuator with relatively constrained radial deformations.

**FIGURE 7:**
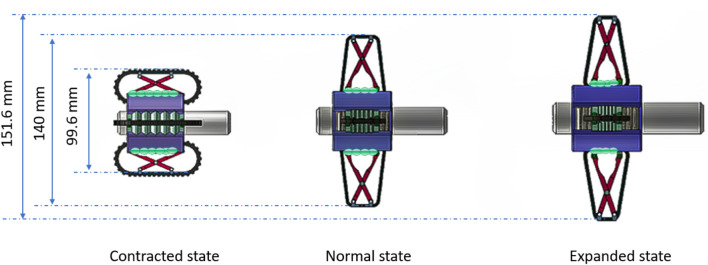
Schematic diagram of working principle of expansion mechanism. Left - bellows are subjected to negative pressure; Middle - bellows are at atmospheric pressure, Right - bellows are subjected to positive pressure.

The four groups of expansion mechanisms are independently controlled by adjusting the air pressure in the bellows, allowing adaptation to the colon’s varying size and shape. The bellows serves as the core component of the expansion mechanism, functioning as a linear actuator. By inflating or deflating the bellows, its volume expands or contracts accordingly, thereby driving a pair of scissor-like supports to move in the vertical direction. This process alters the position of the track and ultimately adjusts the size of the prototype’s head. The prototype is designed to be four times larger than its intended final size (4:1scale), with a non-pressurized diameter 140 mm. The experimental data, which are the average value of ten repeated measurements, indicate that activating the expansion mechanism changes the diameter by almost 53%, from 99.6 to 151.6 mm. When the bellows face negative pressure, they contract, making the diameter of the track frame larger than its original size. Conversely, inflation of the bellows with positive pressure causes the expansion mechanism to contract, as illustrated in Figure 7.

### 3.2 Prototype testing

#### 3.2.1 Testbed

A straightforward experimental platform was established to evaluate the prototype’s performance. The testbed comprises several key components: a 3D-printed pipe support made from PLA, a transparent acrylic pipe, a force sensor, and the prototype system itself. The testbed uses a rigid acrylic tube to mimic a straight section of the colon. This was used to test the propulsion and expansion capabilities of the prototype. While this setup does not replicate the flexibility and complexity of the human colon, it is effective for testing the prototype’s propulsion and expansion capabilities and provides essential data to validate the concept.

The functionality of the prototype was evaluated on a testbed of matching dimensions. To investigate different traction forces, we tested three surfaces: a commercial artificial bowel tissue sheet (Bowel-9mm, Lifelike, Canada), a transparent acrylic tube simulating the colon, and modified versions of the tube’s inner surface. A 3D-printed bracket was used to secure the tube and prevent deviation during testing, while the friction coefficient of the tube’s interior was varied by applying tissue and foam.

**FIGURE 8:**
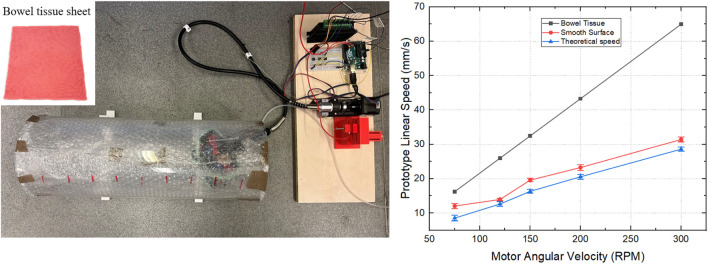
Locomotion experiment of the prototype. The motor angular velocity was set to 75, 120, 150, 200 and 300 RPM to explore corresponding linear velocity of the prototype; Different friction coefficients of the tube surface were emulated using foam, artificial tissue (made of silicone, with mucus) and smooth surface.

Figure 8 compares the theoretical and tested linear velocities of the prototype on various surfaces. Due to friction losses between the flexible shaft, worm gear, tracks, and the tube surface, the observed velocities are lower than the theoretical predictions that do not consider friction. The experimental data are the average of five repeated measurements. Although the prototype reaches maximum speeds of 32.04 mm/s on a smooth surface and 29.29 mm/s on wet tissue at the motor speed of 300 RPM, such speeds may be impractical for operation inside the human colon. It is important to note that these figures demonstrate the prototype’s maximum capabilities in its oversized version. For practical application, motor speed can be precisely controlled to ensure a more appropriate and safe movement speed within the colon, thus ensuring effective operation without compromising patient safety.

#### 3.2.2 Prototype propelling force

In an ideal scenario, the motor’s resisting torque is assumed to counteract only the frictional forces between the track and the worm gear, disregarding friction in the flexible shaft and other forms of power loss. From the motor’s torque-speed curve, we can see that, within the 75–300 rpm range, the motor’s output torque remains nearly unchanged. Under ideal conditions, this minor variation can therefore be disregarded, and the prototype’s theoretical propulsion force can be derived using the appropriate formula. To further validate this propulsion force under varying rotational speeds and pipeline friction coefficients, we developed a dedicated test platform specifically designed to measure its propelling performance.

**FIGURE 9:**
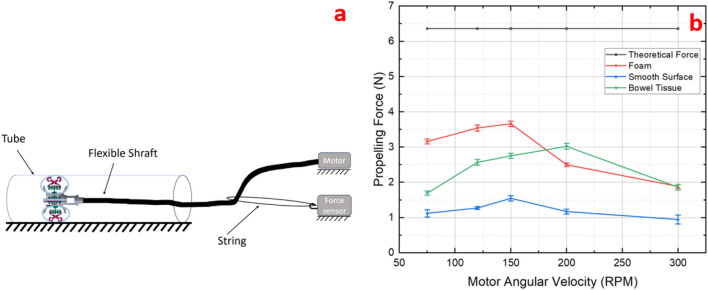
**(a)** Propelling force experiment schematic diagram; **(b)** Comparison of different propelling forces as a function of motor speed measured on three different surfaces with varying friction coefficients and surface hardness.

The force sensor (LSb201, FUTEK, United States) is mounted on the platform and connected to the flexible shaft with a string to measure the propelling force of the prototype. We evaluated the change in propelling force on three surfaces with different friction coefficients—smooth tube, foam, and artificial bowel tissue—by varying the motor speed from 75 to 150 RPM across five sets of repeated experiments, as depicted in Figure 9b. In the range of 75–150 RPM, the force changes as anticipated; however, an increase in speed beyond 150 RPM results in a decrease in propelling force. This decrease can be attributed to reduced transmission efficiency of the flexible shaft at higher speeds, affecting the coupling between the worm and the track and leading to slippage. The experimental results show that the maximum propelling forces achieved are: 1.47 N on the smooth surface, 2.83 N on artificial bowel tissue, and 3.61 N on foam surface. Notably, the device exhibits increased stability on artificial bowel tissue during testing, which may be attributed to the softer material’s ability to undergo concave-like deformations, thereby enlarging the contact area with the device. This observation suggests that the mechanical properties of the testing materials can significantly influence the performance of the prototype. However, further detailed investigation is necessary. This should include a comprehensive study of material properties and their impact on the device’s interaction with its environment.

#### 3.2.3 Experiment of diameter size-adapting

**FIGURE 10:**
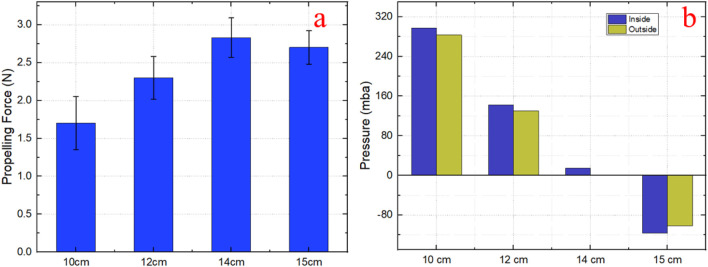
Effect of pipe diameter on bellows pressure and propelling force in a prototype expansion mechanism. **(a)** Expansion mechanism in nominal state; **(b)** Conditioned maintenance of stable traction force.

In this experiment, four different tube diameters (100, 120, 140, and 150 mm) were used to verify the shape adaptability of the prototype. The previous results indicated that the prototype achieves maximum propulsion when the motor angular speed of the is 150–200 RPM, as shown in Figure 9b. To maintain consistency, the motor rotation speed was set at 200 RPM for all tests. The inner surface of the large plastic tubes was lined with artificial tissue to closely simulate the colon’s surface. With these controlled parameters, we measured variations in the propulsion force of the prototype as it navigated tubes of different diameters, as depicted in Figure 10.

As the prototype traverses a smaller tube, the traction force decreases with the reduction in pipe diameter, despite constant air pressure in the bellows. This decrease is attributed to an increase in normal force exerted between the pipe wall and the expandable tip, which in turn places greater force on the struts. Given that the struts are integral to the mechanism’s expansion, this higher normal force consequently impacts the bellows’ performance. To understand this dynamic further, we investigated how the pressure within the bellows varied across four different pipe diameters.

In Figure 10b, the blue bars labelled “inside” represent the average pressure that the bellows must maintain to achieve consistent propulsion force. Conversely, the yellow bars, labelled ‘outside,’ depict the theoretical pressure required by the prototype without any external constraints. There is a significant difference in air pressure between these two conditions, as demonstrated by the distinct blue and yellow bars.

This discrepancy occurs when the expanding tip needs to overcome both its own weight and the stress exerted by the pipe’s normal forces, particularly when the pipe’s diameter is smaller than the expandable tip’s normal diameter. Consequently, the expansion mechanism must generate additional air pressure to counterbalance these forces and maintain traction. In its normal state, the diameter of the expandable tip is 140 mm, with the bellows’ air pressure at ambient levels, which is represented as 0 in Figure 10b. Even in this state, there is a positive pressure within the bellows that counteracts the weight of the expandable tip.

## 4 Discussion

In this paper, we experimentally determined the propelling force of the prototype under various surface roughness conditions but did not measure the traction force. Propelling force drives the entire prototype, while traction force is exerted by end-effectors (wheels, tracks). In this paper, the traction force is influenced by the friction coefficient between the tracks and the contact surface, as well as the normal stress on the tracks. Research indicates that a traction force of at least 1 N is required to reliably drive the robot (Dogramadzi, 2001). Each wheel of the Rollerball robotic endoscope generates about 0.33 N (Norton et al., 2016), and the SoftSCREEN achieves up to 2 N (Valdastri et al., 2012). In our experiments, the maximum propelling force obtained on the artificial bowel tissue was 3 N. But propelling force and traction force are different in a strict sense, which means that the traction force of our prototype may not be higher compared to other models. Therefore, in the next step, we plan to study the relationship between traction force and propelling force by adjusting the friction coefficient of the track and increasing the normal stress on the track. Besides, the excessive force exerted on colon will cause mucosal damage. Therefore, it is also of great significance to carry out experimental research on traction force to evaluate the safety of the device.

We are also very concerned about the stress and potential damage risks to the colon wall. In subsequent work, we will conduct in-depth evaluation of the safety of the device through *in vitro* tissue experiments and animal model studies, including measuring the pressure and friction exerted by the device on the intestinal wall under different propulsion speeds and bending conditions. We plan to introduce more flexible components in the design and combine it with a real-time sensor feedback mechanism to ensure that it can stop or withdraw in time when abnormal stress is detected, thereby effectively reducing potential damage to tissues while taking into account efficient propulsion, laying a safe and reliable technical foundation for future clinical applications.

We established that the diameter of the colon variability is approximately 73%. The expansion rate of our prototype is about 53%, while SoftSCREEN prototype (Consumi et al., 2023) can extend almost 70% from its nominal diameter. From a propulsion perspective, we initially evaluated the feasibility of miniaturizing the prototype. According to (8), the size of the worm is the main factor influencing the propulsion magnitude. When the diameter of the worm gear is reduced to half of the current size (2:1, twice the target size), with the diameters of other parts also reduced in proportion, the prototype achieves a propulsion force of approximately 9.7 N, while the propulsion force at the final target size is about 16.7 N. It is important to distinguish between propulsion and traction forces. Propulsion force is generated by the worm gear to drive the robot forward, while traction force is the effective force transmitted to the environment, crucial for movement in curved colon sections. As the prototype size decreases, traction force may significantly reduce due to smaller contact areas and greater friction sensitivity, particularly in bends, potentially limiting the robot’s mobility. In practical applications, frictional losses and contact stability are expected to reduce the actual propulsion. Nevertheless, the potential for further miniaturization remains promising. Generally speaking, smaller prototype size has higher expansion rate at the same elongation. In our prototype, the expansion rate is primarily determined by the length of the bellow and the struts. Although, it remains uncertain whether the size of prototype correlates with traction force, optimizing the structure of expansion mechanism and miniaturizing the prototype are potential solutions to improve the expansion rate. We aim to develop a theoretical scaling model with dimensionless parameter analysis for accurate prototype miniaturization, using finite element modelling to analyse the mechanical behaviour across the scales, ensuring functional integrity and clinical viability.

In order to simplify the prototype analysis, a rigid testbed was used that does not emulate flexible colon walls. This allowed us to focus on the internal mechanism variables and less so on the interaction with the deformable environment. This setting admittedly poses limitations since the flexible colon wall would affect the contact area with the tracks and therefore the traction force of the mechanism.

In the propulsion testing experiment, from the perspective of operator, the tip the prototype has a more stable movement compared to other friction coefficient surfaces, and from the results, the setting of 200 RPM in Figure 9b) obtains higher propulsion force. The potential reason is that the soft surface, because of its greater deformation trend, enhances the traction and is conducive to the smoother movement of the prototype. We need to do more in-depth experiments to reveal the relationship.

## 5 Conclusion and future work

In this paper, we propose a design for a shape-adaptive robotic endoscope equipped with an expansion mechanism, aimed at achieving consistent traction across varying colon diameters. The prototype’s expansion mechanism comprises four groups of bellows and struts, each of which can be independently controlled by adjusting the bellows’ pressures. This design significantly enhances the prototype’s flexibility, enabling it to accommodate more complex asymmetric changes and perform advanced functions, such as navigating through the tortuous bends of the colon. While this capability is not explored in the current study, it will be a focus of future research. The experimental results from tests in a simulated colon environment confirm the design’s effectiveness, demonstrating robust propulsion performance and adaptability to different diameters.

Future work will extend beyond merely optimizing the expansion mechanism to include enhancing the robot’s expansion rate. Subsequent research will be based on a model that is closer to the actual colon environment, and will explore in depth the dynamic behaviour of the prototype in this environment. We will use finite element modelling (FEM) to further investigate the deformation of the colon wall under the robot’s load (Evans et al., 2025). This will involve simulating the traction force and exploring the relationship between the tracks’ material properties and the resulting traction inside the colon. Additionally, testing and evaluating the robot’s steering functionality is critical. We plan to verify this feature by using pipes with various angles to simulate different navigational challenges. Moreover, future research will explore the integration of sensors into the robot system to enable autonomous adjustment of functions, such as the expansion mechanism. As for the prototype size, future work will focus on gradually reducing the size of the robot prototype. We will continue to refine the design to ensure it retains necessary functionality while also adapting to actual colon testing and clinical applications.

In summary, the robotic endoscope designed in this study demonstrates significant potential for use in colonoscopy. With further clinical trials and technical optimizations, this innovative design is anticipated to find widespread application in medical practice, enhancing the efficiency and safety of colonoscopy procedures. Ultimately, it could provide substantial support for the early detection and prevention of intestinal diseases.

## Data Availability

The raw data supporting the conclusions of this article will be made available by the authors, without undue reservation.
